# Atrial Myxoma: Presenting as a Large Splenic Infarction

**DOI:** 10.7759/cureus.60505

**Published:** 2024-05-17

**Authors:** Ayesha Khalid, Shehnaz Wasim, Alan Kaell, Lev Lubarsky

**Affiliations:** 1 Internal Medicine, Mather Hospital Northwell Health, Port Jefferson, USA; 2 Rheumatology, Mather Hospital Northwell Health, Port Jefferson, USA; 3 Cardiology, Stony Brook University Hospital, Stony Brook, USA

**Keywords:** asymptomatic myxoma, carney syndrome, transthoracic echocardiogram, splenic infarction, large atrial myxoma

## Abstract

Cardiac myxomas are the most common benign primary heart tumors, with the majority occurring in the left atrium. Clinical manifestations are a result of constitutional, obstructive, and/or embolic events. Complications include myocardial infarction and stroke, as well as renal and limb ischemia. Our unusual case is a middle-aged female who presented with a one-week history of progressively worsening abdominal pain and was found to have a large splenic infarction on a CT scan. There was no personal or family history of autoimmune diseases or hypercoagulable states. The evaluation revealed a large left atrial myxoma confirmed on biopsy after surgical resection. Our patient’s clinical presentation was relatively benign compared to the size of her mass. Although her myxoma was very large, morphologically solid, and attached to the interatrial septum, she did not have any evidence of congestive heart failure. The tumor’s irregular surface and mobility likely led to splenic embolization. Hence, the differential diagnosis of splenic infarction should include left atrial myxoma.

## Introduction

This article was previously presented as a poster at the NYACP Poster Competition on October 28th, 2023. Cardiac myxomas account for 50% to 85% of all primary benign cardiac tumors, with an annual incidence of 0.5 to 1 case per million individuals [1]. They are usually present in middle age with a female predominance. Two different morphological types are evident on the echocardiogram: solid or papillary. Papillary myxomas have a greater potential for embolization, while solid tumors have a higher prevalence of congestive heart failure (CHF) [2]. The majority of myxomas are sporadic, with 75% occurring in the left atrium (LA) [3]. Clinical manifestations are characterized by a triad of constitutional, embolic, and obstructive events based on the size, location, and mobility of the tumor [4]. A splenic infarction is uncommon as an initial presentation. 95% of LA myxomas are revealed by a transthoracic echocardiogram (TTE), but smaller myxomas of the LA may require a transesophageal echocardiogram (TEE) [5]. Once diagnosed, the tumor is surgically resected, given the risk of life-threatening intra- and extra-cardiac complications. 

## Case presentation

A 44-year-old female with no significant past medical history presented to the Emergency Department (ED) with severe epigastric pain associated with nausea and vomiting. The patient reported that her pain started about a week before her presentation. It was dull, intermittent, progressively worsening, and often radiating to the back. On the morning of her arrival at the ED, the patient was driving when she started having sudden-onset, severe pain (8/10 on the pain severity scale associated with nausea) and three episodes of vomiting, which prompted her to seek medical attention. The patient denied any history of smoking or illicit drug use. She last used oral contraceptives one year ago. There was no personal or family history of autoimmune diseases or hypercoagulable states.

**Figure 1:**
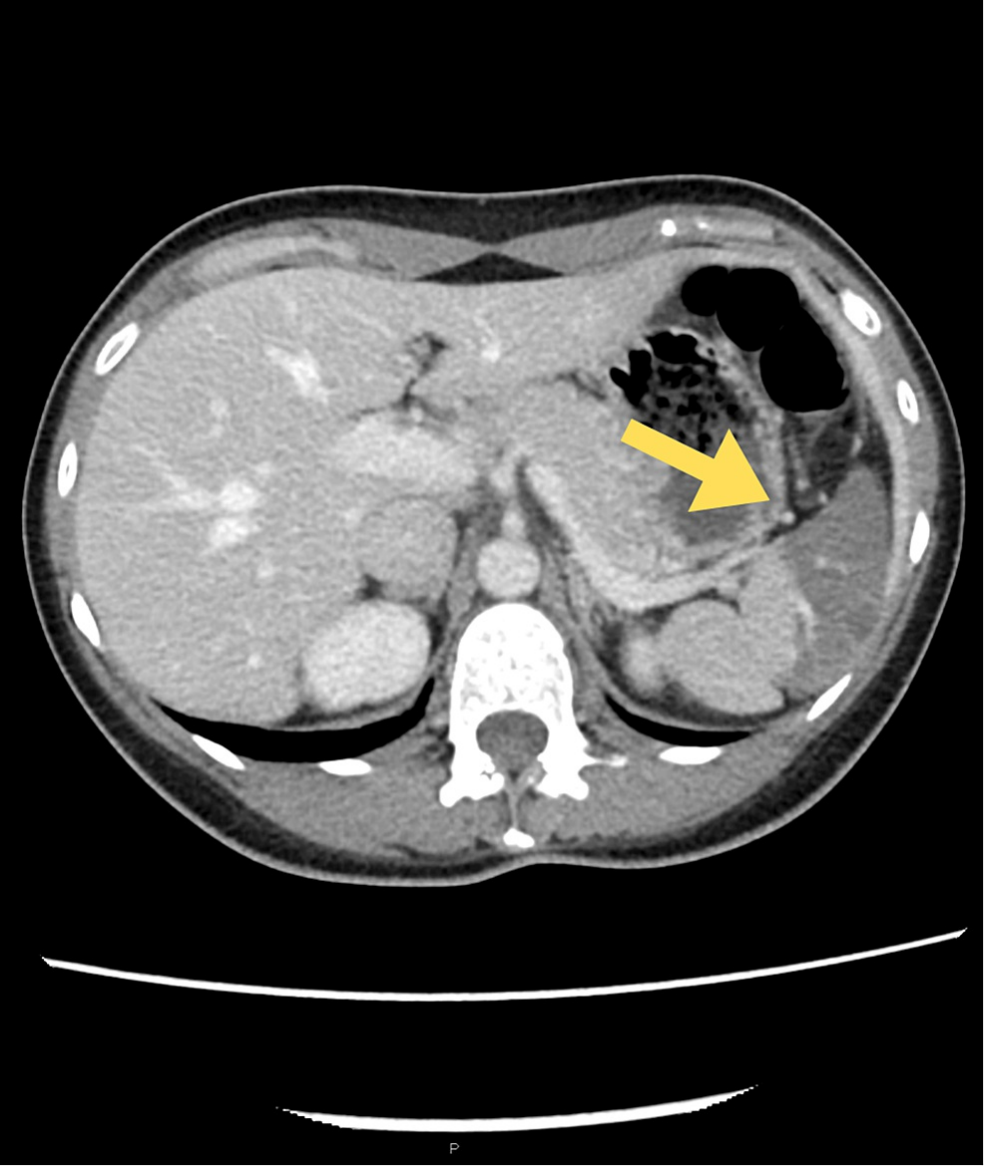
CT abdomen and pelvis with IV contrast showing a large splenic infarction

The patient had a temperature of 97.9℉ with a heart rate of 79 beats per minute and a blood pressure of 115/74 mmHg. On physical examination, the patient’s abdomen was soft, with mild tenderness to superficial and deep palpation in the mid-epigastric region. The rest of her examination was unremarkable. Her laboratory workup showed a white cell count of 20.37 K/uL (ULN = 10.5 K/uL) with a neutrophil predominance of 82% (ULN = 77%). Other labs, including the serum pregnancy test, acute phase reactants, and comprehensive metabolic panel, were normal (Table 1). Urinalysis revealed moderate blood in the urine with a red blood cell count of 21-50 HPF (normal: 0-5 HPF). CT abdomen and pelvis with IV contrast showed a large segmental area of diminished splenic enhancement consistent with splenic infarction (Figure 1). Workup for thrombophilia risk factors was negative (Table 2).

**Table 1:** Laboratory workup on admission INR: international normalized ratio; APTT: activated partial thromboplastin time K/uL: kilo per microliter; %: percent; g/dL: grams per deciliter; mm/Hr: millimeters per hour; sec: seconds; mg/dL: milligrams per deciliter

HEMATOLOGY	PATIENT’S LABS	NORMAL REFERENCE RANGE
White blood cells	20.37 K/uL	3.8 to 10.5 K/uL
Neutrophils %	82.8%	43 to 77%
Hemoglobin	12.6 g/dL	11.5 to 15.5 g/dL
Hematocrit	38.1%	34.5 to 45%
Platelet	337 K/uL	150 to 400 K/uL
Sedimentation rate, erythrocyte	8 mm/Hr	0 to 20 mm/Hr
COAGULATION		
Prothrombin Time	12.9 sec	10.4 to 12.8 sec
INR	1.13	0.91 to 1.12
APTT	29.7 sec	25.5 to 36.5 sec
Fibrinogen	356 mg/dL	210 to 480 mg/dL

**Table 2:** Work up for thrombophilia risk factors dPT: dilute prothrombin time; IgG: immunoglobulin G; IgM: immunoglobulin M; IgA: immunoglobulin A Sec: seconds; GPL: IgG phospholipid unit(s); U/mL: units per milliliter; MPL: IgM phospholipid unit(s); APL: IgA phospholipid unit(s); AT: anti-thrombin; %: percent

TEST NAME	PATIENT’S RESULTS	NORMAL REFERENCE RANGE
Factor V Leiden Mutation	Not Detected	-
dPT	38.6 sec	0.0 to 47.6 sec
Thrombin Time	17.7 sec	0.0 to 23.0 sec
Partial Thromboplastin Time	33.7 sec	0.0 to 51.9 sec
Dilute Russell Viper Venom Time	32.9 sec	0.0 to 47.0 sec
Lupus Anticoagulant	Not Detected	-
Anticardiolipin Antibody, IgG	< 9 GPL U/mL	0 to 14 GPL U/mL
Anticardiolipin Antibody, IgM	< 9 MPL U/mL	0 to 12 MPL U/mL
Anticardiolipin Antibody, IgA	< 9 APL U/mL	0 to 11 APL U/mL
AT III Activity	101%	85 to 135%
Protein C Activity	87%	74 to 150%
Protein S Functional Assay	93%	63 to 140%

**Figure 2:**
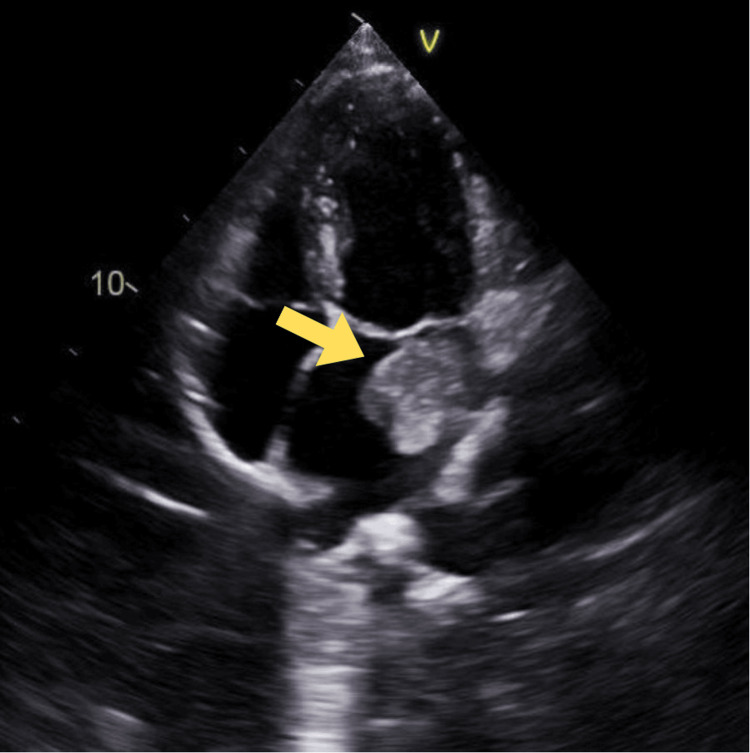
Transthoracic echocardiogram showing a moderately dilated left atrium with a large, irregular 8.3 cm mobile mass in the superior left atrial cavity

**Figure 3:**
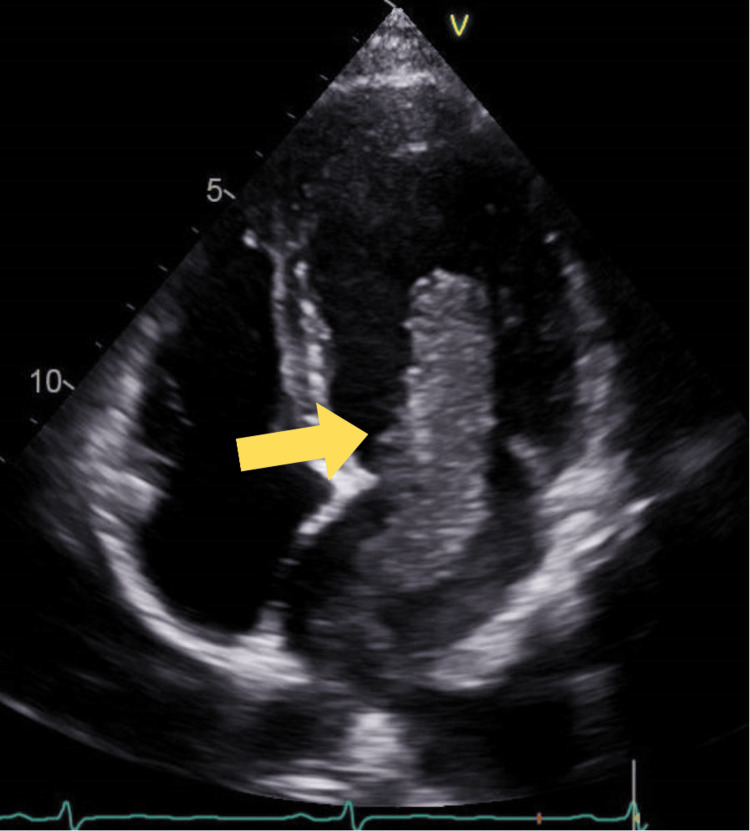
Myxoma can be seen prolapsing through the mitral valve

**Figure 4:**
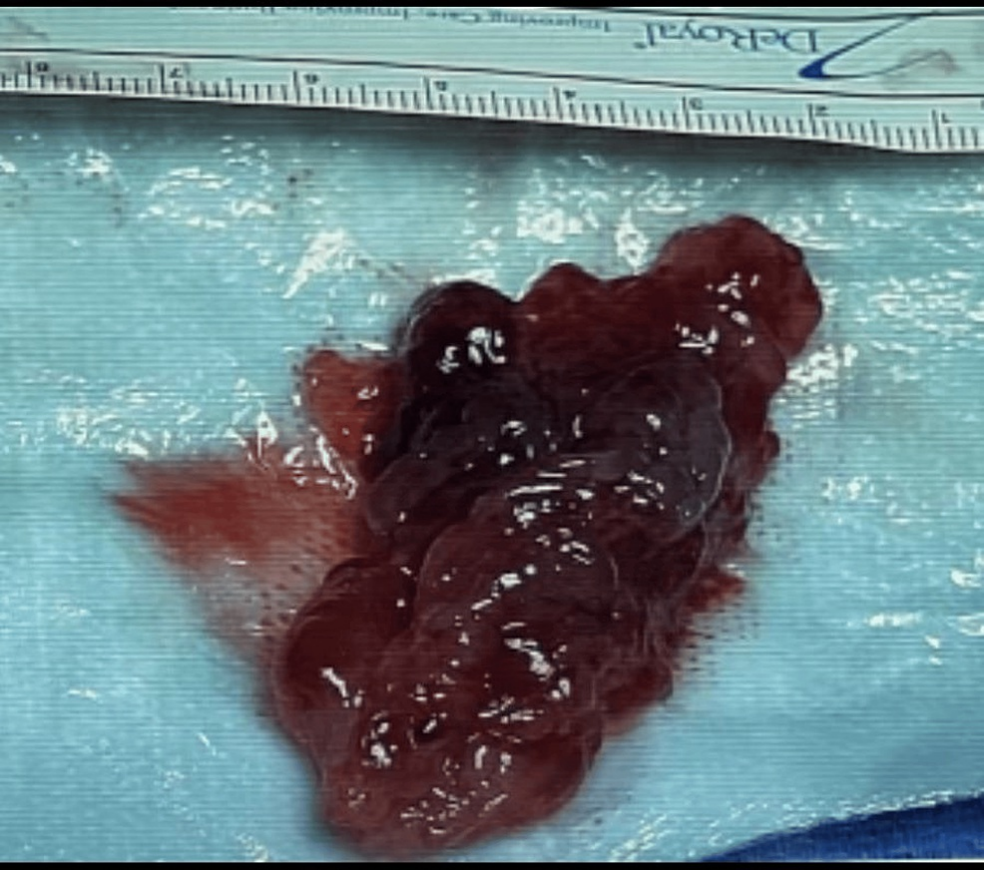
Excised myxoma specimen

TTE showed normal left ventricle (LV) size with an ejection fraction of 61%. LV wall thickness was normal, with no regional wall motion abnormality. The left atrium was moderately dilated with a large, irregular, solid, mobile mass measuring 8.3 cm in the superior left atrial cavity, highly suggestive of an atrial myxoma (Figure 2). Of note, even though the myxoma was seen to be intermittently prolapsing through the mitral valve (Figure 3), the patient did not have a noticeable characteristic murmur called tumor plop on auscultation. Based on the size of her mass, the patient was immediately started on a heparin infusion, and cardiothoracic (CT) surgery was consulted. She underwent resection of the mass via sternotomy (Figure 4). The resected tissue was sent for biopsy, and the results were consistent with myxoma. During follow-up visits at the cardiologist's office, the patient reported feeling well without recurrent symptoms or complications from surgery. 

## Discussion

This case emphasizes the rare manifestation of atrial myxoma in the form of abdominal pain secondary to splenic infarction as the initial diagnosis. This may be a result of different degrees of arrhythmias, intracardiac flow obstruction, and, in our case, the embolic phenomenon involved in the disease process. These varying presentations can occur according to the location, size, and mobility of the mass. It is noted in the literature to have been associated with unusual presentations, including atypical chest pain involving the shoulder and back due to mechanical obstruction of the mitral valve [6]. Knowledge and awareness of this must raise suspicion in clinical practice.

Differentials for a left atrial mass include tumor, thrombus, and vegetation. Echocardiography is a key tool in the investigation of an intracardiac mass. Myxomas usually arise from the fossa ovalis. They are visualized as a mobile mass that is attached to the endocardium by a stalk [7]. If the mass is made of homogenous, well-demarcated, and echo-dense material attached to the left atrial roof, it is more likely a thrombus. This is most often complicated by atrial fibrillation about valvular heart disease. If the mass appears as vegetation around the heart valves or mural endocardium, especially in the setting of diseased native or prosthetic valves, the clinical suspicion would be higher for infective endocarditis. In that scenario, the patient would have constitutional symptoms [8].

Most cases of atrial myxoma are sporadic; however, it may occur in association with an inherited, autosomal dominant disorder called Carney syndrome. This is characterized by multiple extracardiac myxomas, schwannomas, and endocrine tumors [9,10].

Around 30%-40% of patients with atrial myxoma experience symptoms of the embolization of tumor tissue or thrombotic particles mixed with tumor cells [8]. Leukocytosis and fever are common in large-sized myxomas. Studies have shown that interleukin 6 (IL-6) is overproduced in the myxoma tissue and secreted into the systemic circulation, resulting in systemic inflammatory or autoimmune manifestations seen in these patients [11]. Therefore, the need to start these patients on antibiotics is not necessary unless they are demonstrating definite symptoms and signs of an underlying infectious etiology. Outcomes are favorable once successful surgical tumor resection takes place. The recurrence rate is as low as 5% [8]. Cardiovascular magnetic resonance (CMR) is also useful in distinguishing etiology and is, in many instances, superior to echocardiography [6]. A biopsy will confirm the final diagnosis.

This case demonstrated the importance of ruling out cardiac embolic causes as the etiology for splenic infarction in a young woman (Table 3). Although they are mostly benign, presentations may vary, and management includes echocardiography with ultimate surgical resection. 

**Table 3:** Causes of splenic infarction [12] PFO: patent foramen ovale

Causes	Examples
Hemoglobinopathies	Sickle cell disease
Hypercoagulable States	Malignancy, exogenous estrogen use, lupus anticoagulant, protein C and S deficiencies
Hematologic Disorders	Polycythemia vera, leukemia and lymphomas, myelofibrosis
Thromboembolic States	Atrial fibrillation, endocarditis, prosthetic heart valves, PFO
Pancreatic diseases	Pancreatitis
Traumatic	Blunt abdominal trauma

## Conclusions

Splenic infarction is an unusual clinical manifestation of left atrial myxoma. This case signifies the importance of ruling out atrial myxoma as a thromboembolic cause of acute splenic infarction, even if the patient does not demonstrate signs or symptoms of myxoma. Once diagnosed, anticoagulant therapy should be initiated promptly, followed by an urgent resection of myxomatous tissue to prevent life-threatening complications. 
